# Heavy menstrual bleeding on direct factor Xa inhibitors: the MEDEA randomized clinical trial

**DOI:** 10.1016/j.rpth.2024.102448

**Published:** 2024-05-20

**Authors:** Eva N. Hamulyák, Hanke M.G. Wiegers, Barbara A. Hutten, Maria E. de Lange, Anne Timmermans, Peter E. Westerweel, Marten R. Nijziel, Frederikus A. Klok, Marcel M. Hovens, Pieter W. Kamphuisen, Harry R. Büller, Saskia Middeldorp, Luuk J.J. Scheres

**Affiliations:** 1 Department of Vascular Medicine, Amsterdam University Medical Centers, University of Amsterdam, Amsterdam, The Netherlands; 2 Department of Epidemiology and Data Science, Amsterdam University Medical Centers, University of Amsterdam, Amsterdam, The Netherlands; 3 Amsterdam Cardiovascular Sciences Research Institute, Diabetes & Metabolism, Amsterdam, The Netherlands; 4 Department of Gynecology and Obstetrics, Amsterdam University Medical Centers, University of Amsterdam, Amsterdam, The Netherlands; 5 Department of Internal Medicine, Albert Schweitzer Hospital, Dordrecht, The Netherlands; 6 Department of Hematology, Catharina Hospital Eindhoven, Eindhoven, The Netherlands; 7 Department of Medicine – Thrombosis and Haemostasis, Leiden University Medical Center, Leiden, The Netherlands; 8 Department of Internal Medicine, Rijnstate Hospital, Arnhem, The Netherlands; 9 Department of Internal Medicine, Tergooi Hospital, Hilversum, The Netherlands; 10 Department of Internal Medicine, Radboud University Medical Center, Nijmegen, The Netherlands; 11 Department of Clinical Epidemiology, Leiden, University Medical Center, Leiden, The Netherlands

Anticoagulant therapy may lead to abnormal uterine bleeding, including heavy menstrual bleeding, which can significantly impact the quality of life [1,2]. Recent studies indicate that up to 70% of women initiating therapy with direct oral anticoagulants experience heavy menstrual bleeding [1,2]. Clinical practice with regard to management is diverse, with suggested strategies including modification of anticoagulation, addition of tranexamic acid, and/or hormonal therapy [3,4]. In comparison with vitamin K antagonists, direct oral factor (F)Xa inhibitors (rivaroxaban, apixaban, and edoxaban) are associated with an increased risk of abnormal uterine bleeding; however this risk may be lower with dabigatran, a direct oral thrombin inhibitor [2,5]. Although the administration of the antifibrinolytic agent tranexamic acid during the menstrual period may be effective, it has not been prospectively evaluated in women with an indication for therapeutically dosed anticoagulation. Importantly, any available evidence to guide clinical decision making in this setting is derived from observational studies, and no randomized trial evaluating the optimal management strategy for this common clinical scenario has been conducted.

The MEDEA study was a randomized, open-label, pragmatic clinical trial designed to evaluate management strategies in premenopausal women experiencing FXa inhibitor–associated heavy menstrual bleeding (Netherlands Trial Register: NL7760). The rationale and study design have been published previously [6]. In summary, the trial included women on FXa inhibitor therapy at any dose and for any indication presenting with heavy menstrual bleeding as evidenced by a single pictorial blood loss assessment chart (PBAC) score of at least 150. Additionally, participants needed to have an indication for anticoagulant treatment for more than 3 months after inclusion. Participants were randomized to 1 of 3 study arms: 1) switch to dabigatran, 2) continue FXa inhibitor with addition of tranexamic acid during the menstrual period, and 3) continue FXa inhibitor without any intervention. The primary outcome was the difference in PBAC scores before (T1) and after randomization (T2, T3, and T4), with each woman serving as her own control. The estimated sample size of 40 women per arm, ie, 120 for the overall trial, was based on a PBAC score reduction of at least 25% with 80% power at a significance level of .05, also accounting for potential loss to follow-up. Participants provided written informed consent and the trial received approval from the Medical Ethical Committee of the Amsterdam University Medical Centers, Amsterdam, The Netherlands.

Between February 2020 and November 2022, a total of 24 women were screened for eligibility, of whom 16 were randomized to 1 of the 3 study arms. The most common reason for exclusion was a PBAC score below 150. The study was stopped prematurely due to the low inclusion rate. Demographics and clinical characteristics of the study population are presented in the Table. Two women did not have any PBAC measurements after randomization; 1 woman in the dabigatran group stopped study participation and 1 woman in the continue FXa inhibitor without intervention group did not have any menstrual period after randomization. The mean difference (95% CI) in PBAC score before and after randomization was −183 (95% CI, −487 to 121) in the 4 women randomized to dabigatran, −199 (95% CI, −343 to −54) in the 5 women who continued the FXa inhibitor with addition of tranexamic acid during the menstrual period, and 30 (95% CI, −336 to 396) in the 7 women who continued FXa inhibitor without intervention (Table; Figure). No serious adverse events were recorded; 1 woman initiated a proton pump inhibitor for acid reflux after switching to dabigatran.

**Table:** Demographic and clinical characteristics of study population.

Variable	Dabigatran^a^*n* = 4	Continue FXa inhibitor + tranexamic acid *n* = 5	Continue FXa inhibitor^b^*n* = 7
Age (y), mean (SD)	38.3 (11.2)	42.0 (6.4)	40.9 (8.4)
BMI (kg/m^2^), mean (SD)	26.0 (6.4)	28.7 (10.7)	37.2 (10.1)
Type of FXa inhibitor at baseline, n (%)			
Apixaban	1 (25)	1 (20)	3 (43)
Edoxaban	1 (25)	0	0
Rivaroxaban	2 (50)	4 (80)	4 (57)
Indication for anticoagulation, n (%)			
Deep vein thrombosis/pulmonary embolism	3 (75)	4 (80)	7 (100)
Cerebral sinus venous thrombosis	1 (25)	0	0
Superficial vein thrombosis	0	1 (20)	0
Anticoagulation duration (d),^c^ median (range)	594 (164 to 1080)	474 (330 to 1302)	174 (37 to 2588)
Comorbidity, n (%)			
Known gynecologic disorder^d^	0	2 (40)	2 (29)
Known bleeding disorder	0	0	0
Concomitant medication, n (%)			
Antiplatelet therapy	0	0	0
Oral contraceptives	1 (25)	0	0
Intrauterine devices	1 (25)	0	1 (14)
PBAC at baseline, mean (SD)	334 (161)	371 (139)	286 (127)
PBAC after randomization, mean^e^ (SD)	150 (133)	173 (84)	316 (418)
Difference in PBAC			
Mean (95% CI)	−183 (−487 to 121)	−199 (−343 to −54)	30 (−336 to 396)
Median (range)	−125 (−324 to −101)	−211 (−320 to −43)	−37 (−372 to 678)

BMI, body mass index; FXa, factor Xa; PBAC, pictorial blood loss assessment chart.

a One woman in the “dabigatran” group stopped study participation.

b One woman in the “continue FXa inhibitor” group did not have any menstrual period after randomization.

c Time between start of anticoagulation and study randomization in days.

d Previously diagnosed gynecologic disorders included adenomyosis (*n* = 2), myomas (*n* = 1), and polycystic ovary syndrome (*n* = 1).

e Mean PBAC score is based on minimal 1 to maximal 3 subsequent menstrual cycles.

**Figure:**
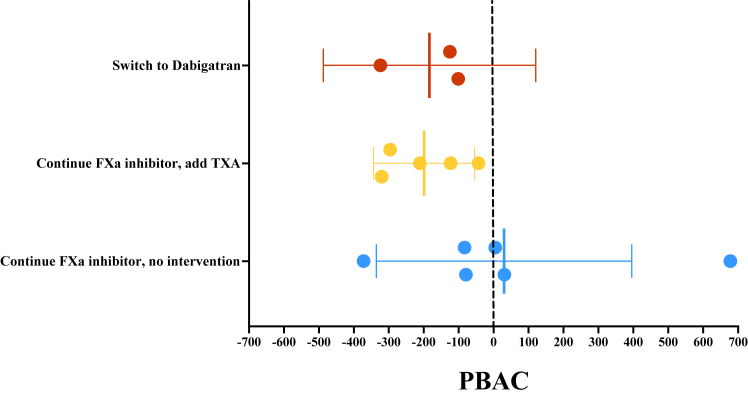
Mean difference in pictorial blood loss assessment chart (PBAC) score at baseline and after randomization. Dots represent the difference in PBAC score at baseline and after randomization for each individual participant by treatment arm. Vertical lines indicate the mean difference by treatment arm (numbers shown in the Table) with corresponding 95% CIs. One woman in the “dabigatran” group stopped study participation. One woman in the “continue FXa inhibitor” group did not have any menstrual period after randomization. FXa, factor Xa; TXA, tranexamic acid.

The MEDEA study was the first randomized trial to assess different management strategies in women with FXa inhibitor–associated heavy menstrual bleeding. A multidisciplinary collaborative effort resulted in a pragmatic clinical trial that was embedded in routine clinical care in 3 academic and 12 nonacademic hospitals. Unfortunately, due to a low recruitment rate, the study was terminated early as the intended sample size of 120 women would not be achieved within a reasonable time frame. While the COVID-19 pandemic played a key role in the slow recruitment rate, we also found the number of eligible participants to be lower than expected.

Several factors may have contributed to this. First, venous thromboembolism in premenopausal women is mainly associated with hormonal factors (pregnancy and use of hormonal contraceptives), ie, indications that typically require short-term (3 months) anticoagulation, making these women ineligible for this study. Second, an entry PBAC score of at least 150 was required for study participation. Even though this cut-off value aligns with the definition of heavy menstrual bleeding in the leading International Federation of Obstetrics and Gynecology guideline [7], this was the most common reason for exclusion. It is likely that menstrual blood loss increases after initiation of anticoagulation but gradually diminishes in subsequent menstrual cycles, which was also reported in the recently published TEAM-VTE study [2]. Moreover, while the PBAC score is designed as a user-friendly clinical tool, alternative instruments, currently under development, could be considered as a primary outcome measure to quantify heavy menstrual bleeding and accurately capture patients’ experiences [8]. Finally, we speculate that despite its frequent occurrence, heavy menstrual bleeding as a potential side effect of anticoagulation may not always be explicitly discussed during counseling or follow-up of premenopausal women who initiate therapy.

What have we learned from the design and execution of the MEDEA study? The entry PBAC score was required to enable a before and after randomization comparison. If we would have chosen to randomize women after the first menstrual cycle following the initiation of anticoagulation, PBAC scores might have been higher and more women would have been eligible. It would likely not have led to an overestimation of the intervention’s efficacy, as the study design also contained a control group without intervention. However, the second measurement prior to randomization was intended to confirm heavy menstrual bleeding and enable the comparison of structurally measured PBAC scores before and after randomization. Furthermore, we observed that many women were promptly referred to a gynecologist after the onset of heavy menstrual bleeding for counseling on hormonal therapy, mainly intrauterine devices but also combined oral contraception while on anticoagulant therapy. These interventions are known to effectively reduce heavy menstrual bleeding and frequently used as treatment thereof, but were not compatible with participation in the current trial. An alternative approach would have been to randomize women at the initiation of anticoagulation to either a direct thrombin inhibitor or FXa inhibitor. In this scenario, women without heavy menstrual bleeding would have been included as well, but it may have provided insights into whether upfront management with a direct thrombin inhibitor could lead to lower rates of heavy menstrual bleeding than those with FXa inhibitors. The RAMBLE trial aimed to evaluate heavy menstrual bleeding associated with rivaroxaban and apixaban for treatment of venous thromboembolism, but has unfortunately also stopped due to low inclusion rates during the COVID-19 pandemic [9].

It was our ambition to evaluate the efficacy of both a switch to dabigatran and addition of tranexamic acid. We hypothesized that switching from an FXa inhibitor to a direct thrombin inhibitor or adding tranexamic acid during the menstrual period would result in a reduction in heavy menstrual bleeding. In both intervention groups, similar numeric trends toward reduced bleeding scores were observed, while the mean bleeding scores remained unchanged in the control group. However, given the premature ending of the trial without meeting the predefined sample size, we are unable to draw solid conclusions from these data. Further studies are urgently needed, given the high incidence and burden of heavy menstrual bleeding in this population. Our findings are hypothesis-generating and support further investigation of both a switch to dabigatran and addition of tranexamic acid during the menstrual period as clinical strategies to mitigate FXa-associated heavy menstrual bleeding. Increasing awareness of this common clinical issue among health care providers is key, as this could lead to tailored counseling of premenopausal women commencing anticoagulation and encourage these women to reach out when experiencing heavy menstrual bleeding.

In conclusion, FXa inhibitor–associated heavy menstrual bleeding is an important clinical problem having substantial impact on quality of life and posing risks of medical interventions and hospitalization. The need for high-quality evidence-based treatment strategies for anticoagulation-associated heavy menstrual bleeding is evident.
